# A Novel Two-Step Technique for Retrieving Fractured Peripherally Inserted Central Catheter Segments Migrating into the Heart or the Pulmonary Artery

**DOI:** 10.1155/2016/7814529

**Published:** 2016-08-23

**Authors:** Juan Peng, Xiao-Ming Zhang, Lin Yang, Hao Xu, Nan-Dong Miao, Yong-Jun Ren, Kang Liu, Xu-Li Min, Ke Yang, Shi Yang, Cheng Yang

**Affiliations:** Sichuan Key Laboratory of Medical Imaging, Department of Radiology, Affiliated Hospital of North Sichuan Medical College, Nanchong, Sichuan 637000, China

## Abstract

*Objective*. To report the experience of a percutaneous technique for retrieving fractured peripherally inserted central catheter (PICC) segments migrating into the heart or the pulmonary artery.* Method*. From April 2013 to July 2015, we performed percutaneous retrieval of fractured PICC segments migrating into the heart or the pulmonary artery in five cancer patients who had undergone chemotherapy via PICC. The fractures were diagnosed with chest plain radiography. The patients included three cases of breast cancer, one case of rectal cancer, and one case of lower limb Ewing's tumor. The fractures were retained in the vessels of the patients for 1 to 3 days. All the fractures were retrieved by using a novel two-step technique in the digital subtraction angiography (DSA) suite. This two-step technique involves inserting a pigtail catheter to the heart or the pulmonary artery to grasp the fractured catheter fragment and bring it to the lower segment of the inferior vena cava, followed by grasping and removing the catheter fragment with a retrieval loop system of the vena cava filter retrieval set.* Result*. The fractured PICC segments were removed successfully in all five patients via unilateral (four patients) or bilateral (one patient) femoral vein access. No complications occurred during the interventional procedure.* Conclusion*. Percutaneous retrieval can be a safe, convenient, and minimally invasive method for the removal of fractured PICC segments. The technique reported in this paper will be applicable for the retrieval of fractured PICC segments and other catheter fragments migrating into the heart or the pulmonary artery.

## 1. Introduction

Peripherally inserted central catheters (PICCs) are widely used to provide central venous access in chronically ill patients with long-term intravenous access requirements, such as those related to chemotherapy, parenteral alimentation, irritant drug infusion, and so forth. Multiple substantial complications of PICC, including catheter malposition, migration, obstruction, infection, thrombosis, and catheter fracture, have been reported in the literature [1–5]. Fractured catheter fragments should be retrieved to prevent further complications. Percutaneous retrieval of intravascular foreign bodies is considered the gold standard treatment because it is a minimally invasive, relatively simple, safe procedure, with low complication rates compared to conventional surgical treatment [6, 7]. However, it can be difficult to retrieve the fractured catheter segments migrating into the heart or the pulmonary artery [8], so advanced techniques are required. In this report, we described our experience of 5 patients with fractured PICC segments in the heart or the pulmonary arteries which were successfully retrieved with a novel two-step method.

## 2. Materials and Methods

### 2.1. Patients

This is a retrospective report based on the medical records of 5 consecutive patients treated from April 2013 to July 2015. Of the 5 patients, 2 were males and 3 were females, with an average age of 39.4 ± 17.2 years (range: 10 to 55 years). The patients included three cases of breast cancer, one case of rectal cancer, and one case of lower limb Ewing's tumor (Table 1). One patient presented cardiac symptoms (frequent ventricular premature beat), and the other four patients were asymptomatic with the fractured PICC segments. The fractured PICC segments were retained in the vessels of the patients for 1 to 3 days. The fracture was diagnosed with chest plain film.

### 2.2. Techniques

Through percutaneous right femoral venous access, the 11 Fr. coaxial retrieval sheath system of a vena cava filter retrieval set (William Cook Europe Aps, Sandet 6, DK-4632, Bjaeverskov, Denmark) was advanced to the inferior vena cava over the wire guide (guide wire 0.035, 180 cm; Terumo Corporation, 44-1, 2-Chome, Hatagaya, Shibuya-ku, Tokyo 151-0072, Japan), and then the inner coaxial catheter and wire guide were removed. The position of the coaxial retrieval sheath system was verified by injecting contrast medium. A 5 Fr. pigtail catheter (straight pigtail 0.038, 110 cm; Terumo Corporation, 44-1, 2-Chome, Hatagaya, Shibuya-ku, Tokyo 151-0072, Japan) was placed at the fractured catheter body over the same wire guide. At the moment when the wire was removed, the fractured catheter body was grasped by the pigtail catheter. After the pigtail catheter and the fractured catheter coiled each other by rotating the pigtail catheter, the fractured catheter was brought to the lower segment of the inferior vena cava to be removed by pulling the pigtail catheter. After the pigtail catheter was removed, the retrieval loop system of the vena cava filter retrieval set was introduced through the coaxial retrieval sheath system to the inferior vena cava, until it was placed at the anterior end of the fractured catheter. At this moment, the fractured catheter was grasped by the device and taken to the sheath to be removed. A follow-up chest radiograph excluded residual fragments in the heart and the pulmonary artery, as well as the inferior vena cava. All the procedures should be performed under ECG guidance systematically.

## 3. Result

**Figure 1:**
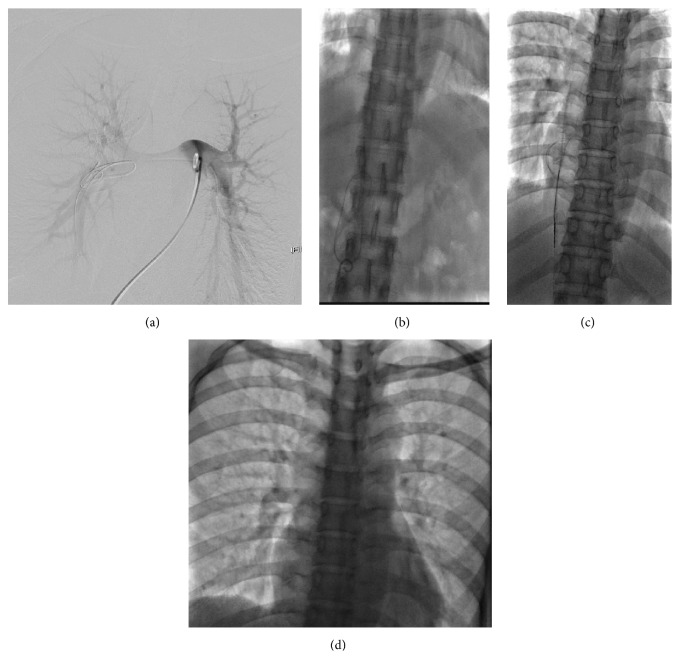
(a) The proximal and distal ends of the fractured catheter were located in the left and right branches of the pulmonary artery trunk, respectively, and the fractured catheter body was grasped by a pigtail catheter. (b) The fractured catheter was brought to the lower segment of the inferior vena cava to be removed. (c) The fractured catheter was grasped by the loop system of the vena cava filter retrieval set to be removed. (d) Chest radiograph after percutaneous retrieval of the fractured catheter, demonstrating the absence of any residual fragments.

In this group of consecutive patients, the location of the fractured catheters was confirmed by an X-ray examination. The proximal and distal end of the fracture were located in the left and right branches of the pulmonary artery trunk, respectively, in two patients, and the proximal end was located in the superior vena cava and the distal end in the trunk of the pulmonary artery in two patients. The proximal end was located in the right atrium and the distal end in the right ventricle in one patient. All the fractured catheters were removed successfully without complications (Figure 1). In all the cases, the right femoral access was used in 4 cases, and both the right femoral access and the left femoral access were used in 1 case. The median of duration time of the procedure is 9 min with a range of 7 to 69 min.

## 4. Discussion

If a catheter fracture occurs, the broken catheter will migrate distally along the blood stream and finally lodge in the superior vena cava, the right atrium, the right ventricle, the main pulmonary artery or its branches. Surov et al. [8] studied a total of 215 cases of intravenous catheter embolization. In their group, sites of catheter fragments were the superior vena cava or peripheral veins (15.4%), the right atrium (27.6%), right ventricle (22.0%), and pulmonary arteries (35.0%). The most common site for fragments was the pulmonary artery (35.0%). In this group, the proximal end and the distal end of the fracture were located in the left and right branches of the pulmonary artery trunk, respectively, in two patients; the proximal end was located in the superior vena cava and the distal end in the trunk of the pulmonary artery in two patients; and the proximal end was located in the right atrium and the distal end in the right ventricle in one patient.

The majority of patients have no or modest symptoms but substantial sequelae may develop. The clinical presentation of catheter embolization varies considerably. A systematic review [8] reported that the clinical signs included catheter malfunction (56.3%), arrhythmia (13.0%), pulmonary symptoms (4.7%), and septic syndromes (1.8%). In this study, only 1 patient presented frequent ventricular premature beat, and the other 4 patients were asymptomatic. Intravascular foreign bodies should be removed to prevent potentially lethal complications. When there are symptoms or the risk of infection is high, the foreign body should be removed promptly. If the catheter adheres to the wall of the right heart system, leading to incessant arrhythmia, or the catheter goes through the unclosed foramen ovale into the left heart system, leading to serious artery embolism, it needs to be removed urgently. Usually, transient arrhythmia (premature beat and tachycardia) related to endocardiac catheter maneuvers may occur but disappears quickly after exiting the catheter.

Several percutaneous transcatheter retrieval techniques including a loop snare, a guide wire, a balloon, a forceps, and a basket catheter have been applied to remove cardiovascular catheter fragments and other foreign bodies [9–27]. The loop snare method is relatively safe with reliable effects, so it is currently widely used [9–11, 15–18]. However, when using a loop snare to remove PICC fractures, the loop snare must be placed at the end of the catheter to grasp it. If the end of the catheter is lodged in the vessel wall or in a difficult plane, it will be difficult to grasp it successfully [19]. If the fractured catheter is located in the pulmonary artery, especially in the pulmonary artery branches, it will also be difficult to successfully grasp the end of the fractured catheter, because the movement of the loop cannot be easily controlled in the pulmonary artery and its branches. Teragawa et al. [9] reported a successful endovascular technique using a snare with a suture to retrieve a migrated broken PICC in the pulmonary artery of a chemotherapy patient. Although their technique is interesting and a useful method to control catheter movement, it may be associated with a risk of vascular injury and other unresolved problems, such as those relating to the thickness and type of suture used. Kawata et al. [10] experienced three cases of retrieval of silicone port catheters migrating into the cardiac ventricle or pulmonary artery. Several devices, including a snare wire, an ablation catheter, and a basket catheter, in combination with an interventional guiding catheter were applied to retrieve them. Yen et al. [11] reported 13 patients who had an embolization caused by central venous catheter fragments including 4 PICCs, and they utilized the “goose-neck” snare to retrieve a catheter fragment with its free end floating in the pulmonary trunk. If the fractured catheter fragment was engaged in the trabecula of the right ventricle and could not be grasped by the loop snare, a floppy guide wire was inserted through the other vein to cross the fragment and grasp its tip using the loop snare. As discussed previously, a guide wire can be used with a catheter to construct a homemade loop snare. The technique of the balloon is useful in the recovery of stents; it requires a guide wire passing through the intravascular foreign body (IFB) or a portion of it. It is important to choose an appropriate retrieval balloon. If the balloon is too large, it will not pass the IFB; if the balloon is too small, it will not capture the IFB [22, 28]. The grasping power of the forceps is advantageous in removing a foreign body strongly adhering to the vessel wall; however, in this case, to control the catheter head is difficult. The catheter material is stiff, and there is a danger of causing damage to vessels [22, 28, 29]. The basket is a well-known device that is often used in the biliary system. This catheter is capable of withdrawing relatively large foreign bodies and is preferred in situations where a foreign body is attached to the vessel wall without a free edge [29]. As it is made of stiff material with less flexibility, it can sometimes damage the vessel wall [22, 28, 29]. Another disadvantage of basket is its poor navigation capability [28].

In the present study, the fractured PICC segments located in the heart or the pulmonary artery were removed successfully using a pigtail catheter in combination with a vena cava filter retrieval set. However, there is a drawback of losing the retrieved catheter during the moment in between the two steps. If the fractured catheter fragment returns to the heart from the inferior vena cava, a pigtail catheter is inserted through the other femoral vein to bring the fractured catheter fragment to the lower segment of the inferior vena cava, and then it can be grasped and removed by the retrieval loop through the coaxial retrieval sheath system successfully. These procedures will increase the duration of the procedure. In this study, both the right femoral access and the left femoral access were used in 1 case; the duration of the procedure is 69 min.

Our experience is that the fractured catheter bodies in the heart or the pulmonary artery can be easily grasped using a pigtail catheter, and when the fractured catheters are brought to the inferior vena cava, they can also be easily grasped and removed using a vena cava filter retrieval set.

In conclusion, a two-step method of retrieving fractured PICC segments migrating into the heart or the pulmonary artery is described in this study. It adds a valuable technical option to the existing percutaneous techniques for retrieving cardiovascular foreign bodies.

## Figures and Tables

**Table 1:** Summary of patient details.

Case number	Sex	Age	Diagnosis	Access
1	Female	44 years old	Breast cancer	Right femoral vein
2	Female	41 years old	Breast cancer	Right femoral vein
3	Female	47 years old	Breast cancer	Right femoral vein
4	Male	55 years old	Rectal cancer	Bilateral femoral vein
5	Male	10 years old	Lower limb Ewing's tumor	Right femoral vein
